# Hyperarousal Is Associated with Socioemotional Processing in Individuals with Insomnia Symptoms and Good Sleepers

**DOI:** 10.3390/brainsci10020112

**Published:** 2020-02-20

**Authors:** Reuben D. M. Howlett, Kari A. Lustig, Kevin J. MacDonald, Kimberly A. Cote

**Affiliations:** Psychology Department, Brock University, 1812 Sir Isaac Brock Way, St. Catharines, ON L2S 3A1, Canada; rh16yq@brocku.ca (R.D.M.H.); kl14qc@brocku.ca (K.A.L.); km11pv@brocku.ca (K.J.M.)

**Keywords:** insomnia, hyperarousal, emotion processing

## Abstract

Despite complaints of difficulties in waking socioemotional functioning by individuals with insomnia, only a few studies have investigated emotion processing performance in this group. Additionally, the role of sleep in socioemotional processing has not been investigated extensively nor using quantitative measures of sleep. Individuals with insomnia symptoms (*n* = 14) and healthy good sleepers (*n* = 15) completed two nights of at-home polysomnography, followed by an afternoon of in-lab performance testing on tasks measuring the processing of emotional facial expressions. The insomnia group self-reported less total sleep time, but no other group differences in sleep or task performance were observed. Greater beta EEG power throughout the night was associated with higher intensity ratings of happy, fearful and sad faces for individuals with insomnia, yet blunted sensitivity and lower accuracy for good sleepers. Thus, the presence of hyperarousal differentially impacted socioemotional processing of faces in individuals with insomnia symptoms and good sleepers.

## 1. Introduction

The capacity to function well in daytime life is dependent on a night of quality sleep. Approximately 10%–15% of the population suffers from chronic poor sleep due to insomnia, a chronic condition characterized by difficulty initiating and/or maintaining sleep [1,2,3,4]. Insomnia leads to daytime performance deficits in cognitive abilities such as memory, attention, and concentration, and several of these performance deficits have been linked to poor sleep [5,6,7,8,9,10,11]. Another feature of insomnia is reports of reduced social engagement [12,13,14], suggesting that one consequence of insomnia is poor social functioning. However, little research has examined the processing of socioemotional content in patients with insomnia, despite complaints of daytime impairment in these areas (e.g., [12,15]), and the literature showing that both experimental sleep deprivation and insomnia lead to alterations and/or impairments in emotion processing (for reviews, see [16,17]).

There is some evidence to suggest a role for sleep in waking emotion processing in insomnia. Less time in SWS and REM sleep has been linked to heightened amygdala reactivity to intense negative emotion stimuli [18]. Moreover, the functional connectivity of orbitofrontal areas (a region of emotion processing) was also positively associated with complaints of poorer sleep history [19]. Waking differences in the activity of the fusiform gyrus, a region associated with the processing of faces, have also been linked to subjective reports of historic poor sleep quality and greater symptom duration of insomnia [20]. Findings of altered relative glucose metabolism, in sleep relative to wake, in regions associated with salience and emotion processing in individuals with insomnia [21,22], suggest that the cortical activation of these regions may continue to be engaged and/or uninhibited during sleep, potentially taxing cognitive resources for next-day emotion processing and functioning. Following from this imaging work, the severity of sleep impairment experienced by individuals with insomnia may be associated with the extent of waking impairment in emotion processing.

Only a few studies have investigated waking emotion processing in individuals with insomnia. Baglioni and colleagues reported that insomnia patients showed greater physiological response to emotional picture scenes using measures of facial EMG and heart rate [23]. Subjectively they rated negative sleep-related stimuli as more unpleasant and arousing. In a subsequent fMRI study, insomnia patients showed a heightened amygdala response to insomnia-related negative picture stimuli, which was interpreted as evidence for a negativity bias towards stimuli of salience [18]. Two recent studies more directly addressed waking socioemotional processing in insomnia by measuring categorization accuracy and intensity judgements when viewing emotionally expressive faces [24,25]. Cronlein and colleagues reported that both patients with insomnia and patients with sleep apnea were less accurate than good sleepers at identifying happy and sad faces. Kyle et al. reported that insomnia patients gave lower intensity ratings than good sleeper controls for faces of fear and sadness. Therefore, complaints of poorer social functioning [12,15] might stem from a reduced capacity of individuals with insomnia to correctly identify or be sensitive to the valence of emotional face expressions, which are important cues for socioemotional functioning [24,25,26,27,28]. Neither study by Kyle et al. or Cronlein et al. found support for a role of sleep in waking performance; however, they only reported basic measures of sleep/wake architecture (such as total sleep time and sleep onset latency) related to performance and did not assess more direct measures of EEG power spectra during sleep/wake periods, which may provide additional insight into relationships between sleep physiology and waking performance.

The contributions of sleep to socioemotional processing in individuals with insomnia have not been examined beyond sleep architecture or self-report measures. The limitations of using PSG for quantifying sleep in insomnia have been well documented [29,30]; quantitative EEG measures of sleep offer more sensitive measures of possible alterations in neurophysiological activity in insomnia sleep [31,32,33,34,35,36,37,38,39,40,41,42]. Several studies have reported greater power in the high-frequency beta (16–35 Hz) and gamma EEG bands (40–70 Hz), which are normally predominant in waking brain wave activity [43] during both sleep onset and non-REM sleep in individuals with insomnia compared to good sleepers [32,35,36,37,38,40,41,42]. Elevated high-frequency EEG activity in patients with insomnia is thought to represent abnormal arousal and enhanced information processing during sleep and a misperception about sleep states. As such, the presence of high-frequency EEG in sleep in patients with insomnia has been described as hyperarousal, underlying the etiology and pathophysiology of the sleep disorder (see [5,39,44,45,46] for comprehensive reviews on the topic). Quantitative EEG measurement of sleep allows for the investigation of markers of disturbed sleep beyond differences in gross sleep architecture or subjective perceptions of sleep quality.

The central aim of this study was to examine the relationship between sleep physiology and waking emotion processing in patients with insomnia symptoms compared to good sleepers. To do this, we employed one task that measured the ability to accurately categorize emotionally expressive faces and intensity ratings of those faces using a task previously employed in insomnia patients by Kyle et al. [24], and another emotional face processing task that included an additional element of attentional control to increase the cognitive complexity of the task, particularly given that attentional control has been found to be impaired in insomnia [9,47]. Based on previous findings of differences in salience assessment and perceptual accuracy of emotion expressions [24,25] as well as deficits in attention and inhibitory control in insomnia [5,9], we predicted that individuals reporting insomnia symptoms would have poorer accuracy and lower intensity ratings for emotional face expressions, as well as poorer inhibitory control for emotion distractors. We measured sleep quantitatively using an EEG power spectral analysis and an innovative measure of sleep-depth called the Odds-Ratio-Product (ORP; [48]) in order to more precisely assess the role of sleep physiology in emotion processing. Greater levels of high-frequency EEG activity throughout sleep (e.g., [36,39,41]) and findings of altered relative glucose metabolism in sleep/wake states in regions associated with emotion processing in individuals with insomnia [21,22] suggest that regions of emotion processing may have uninhibited/maintained wake-like activity throughout sleep which could lead to a lack of restoration and/or alteration in waking socioemotional processing the following day. Therefore, we expected that measures of disrupted sleep and hyperarousal over the night would be associated with performance differences on emotion processing tasks for individuals with insomnia symptoms.

## 2. Method 

### 2.1. Participants

Participants with insomnia (INS) met the DSM-5 criteria for diagnosis [49] as well as having an Insomnia Severity Index (ISI; [50]) score greater than seven (i.e., subclinical or greater levels of insomnia symptomology). Eligible INS individuals reported less than 6.5 h of sleep per night on average, greater than 30 min to fall asleep and/or stay awake throughout the night or early morning awakenings without being able to return to sleep, sleep disturbances for at least three nights a week and lasting three or more months, and that their sleep had negative consequences on daily functioning and/or quality of life. These individuals are described herein as having insomnia symptoms because they met the DSM-5 criteria for insomnia, but PSG recording was not carried out to exclude sleep apnea, restless leg syndrome or periodic limb movement disorders (although they did not endorse symptomology on questionnaires). All the participants reported having no other sleep disorders (e.g., sleep apnea, restless leg syndrome), having no psychiatric illnesses (e.g., schizophrenia, depression, anxiety), no history of concussion or head injury, and taking no medication that affects cognition or sleep. They also reported no recent history of trans-meridian travel, shift-work, or smoking. 

Of the 45 participants that passed the preliminary screening and completed the in-lab orientation session, two participants were withdrawn due to technical difficulties with the sleep equipment during the nights of home recording (i.e., no PSG data recorded), one good sleeper was omitted from final analysis for failure to comply with instructions to wake up by 8:00 a.m. on the performance testing day and two participants from the INS group were excluded due to inability to remain awake during performance assessment (i.e., no performance data). One good sleeper was removed who scored greater than seven on the ISI, one good sleeper was removed due to an atypical poor night of sleep, and nine good sleepers were removed because they did not comply with the study instructions and severely restricted their time in bed. Fourteen individuals with insomnia symptoms (INS) (10 women, *M* age = 27 years, *SD* = 9.81) and fifteen good sleeper (GS) controls (13 women, *M* age = 25.60 years, *SD* = 9.56) were included in the data analysis. Participants were paid an $60 honorarium for study completion; the INS group received a copy of a home treatment module [51].

### 2.2. Materials

**Face emotion categorization and intensity task (FCI)**. This task was adapted from the study by Kyle et al. [24]. A variable inter-stimulus fixation point was displayed for 500–800 ms before a face expressing one of four emotions (happy, sad, fearful, angry) was presented for 400 ms. An empty black screen followed each face stimuli and continued until participant response. Participants responded by a keystroke (keyboard number row 1–4) corresponding with each emotion to categorize the face. Participants were then presented, until response, with a 5-point Likert scale to rate the intensity of the face (1 = “Not very intense” to 5 = “Extremely intense”) without the face stimuli present. There was a total of 60 trials of each emotion (240 total trials) and the sequence of stimuli presented was randomized. Stimuli were static greyscale face images (17 cm high) from the NimStim face database [52] and were cropped to remove clothing, background, and hair. This task differed from the one employed by Kyle et al. [24] as follows: different face stimuli, stimulus presentation duration, and response option for intensity ratings was changed from 1–7 to 1–5 as piloting revealed a tendency for participants to not move the hand further along the keyboard to choose higher intensities.

**Face-word emotion Stroop task (EST).** This task was adapted from the study by Preston and Stansfield [53]. A variable inter-stimulus fixation point was displayed for 500–800 ms before a face of one of three emotions (happy, sad, angry) or neutral expression was displayed for 400 ms. Each face stimuli had the words “happy”, “sad”, “angry” or “neutral” displayed across the ridge of the nose in order to not obscure facial or eye cues and to appear in the center where the fixation point was previously located. After 400 ms, the stimulus was removed; the task did not proceed to the next trial until the participant responded. The participant responded by indicating the emotion of the face, not the word (using numpad keyboard keys 0–3), as this approximates the original Stroop task where processing of the semantics of the word is automatic and distracting, requiring inhibitory control [54]. There was a total of 36 congruent (face-word matched) and 36 incongruent trials (face-word did not match) for each face type (288 total trials) and the sequence of stimuli presented was randomized. Stimuli were greyscale images (17 cm high) taken from the Karolinska Directed Emotional Faces database [55] and were cropped to remove clothing, background, and hair. This task differed from Preston and Stansfield [53] as follows: different face stimuli, stimulus presentation duration, using the prototypical emotion words (“happy”, “sad”, “angry”) instead of emotion adjectives (e.g., “blissful”, “hopeless”, “enraged”) as the distractor, and the inclusion of a neutral condition (“neutral” word and neutral face expressions).

Sleep recording equipment. Ambulatory PSG was employed using the Prodigy Sleep System (Cerebra Medical Inc., Winnipeg, Canada) in order to capture more naturalistic sleep in participants’ homes. The Prodigy Sleep System had eight recording channels: left-frontal EEG and right-frontal EEG, ground and reference, all fixed on the forehead in a box, and drop down electrodes for left EOG, right EOG, right mastoid (M2/A2), and left chin EMG. These channels were used to obtain measures of sleep architecture, average EEG power (µV^2^/Hz) for each frequency band over the night, and values of the ORP, a continuous measure of sleep depth ranging 0–2.5 (0 = deepest sleep, 2.5 = awake; [48]).

### 2.3. Procedure

**Figure 1:**
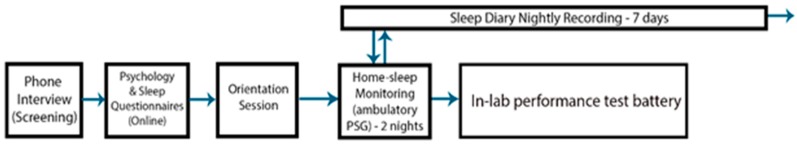
Overview of the progression of study protocol from recruitment to completion.

All the study procedures were cleared by the Brock University Bioscience Research Ethics Board (file#17-322, approval date: 3/28/2018) and the study was conducted in accordance with the Canadian Tri-Council Policy Statement: Ethical Conduct for Research Involving Humans (TCPS2). All subjects gave both verbal and signed consent to participate in the study. For an overview of the study procedure, see Figure 1. Participants were recruited via advertisements on campus and in the community. Individuals interested in the study consented to participation in a preliminary telephone interview to ensure that eligibility criteria were met. Next, they completed a written consent form and additional online questionnaires to further assess eligibility and to collect data on sleep and affect. The subset of the questionnaires reported in the current study included the Pittsburgh Sleep Quality Index (PSQI; [56]) and the Insomnia Severity Index (ISI; [50]), widely used measures of subjective sleep quality, over the past four and two weeks, respectively. The Depression Anxiety Stress Scale (DASS; [57]) and State Trait Anxiety Index–trait (STAI-T; [58]) were completed in order to assess the presence of affective symptomology.

Eligible participants came to the Sleep Research Laboratory for an orientation session where they signed informed consent for laboratory testing and at-home sleep recording and completed a practice version of the performance assessment battery. They were then instructed on use of the Prodigy home sleep monitoring equipment which they used for two consecutive nights at home. Additionally, participants completed an online sleep diary following the first night of PSG recording and for up to seven days after. Immediately following the second night of at-home PSG recording, participants came to the lab at 13:00 and had EEG recorded during a performance assessment battery from 14:00 to 16:00. The battery consisted of a number of information processing tasks, not all reported here. In addition to the two emotion processing tasks reported herein, participants also completed a Psychomotor Vigilance Task (PVT), the Stanford Sleepiness Scale (SSS; [59]), the Positive and Negative Affect Schedule (PANAS; [60]), and the STAI-State questionnaire [58], all commonly used to verify changes in waking function due to insufficient sleep. Upon completing the task battery, electrodes were removed and participants were free to leave the lab after debriefing.

### 2.4. Data Analysis

Sleep files were first scored by automatic sleep-scoring software (Michele software, [61]) following AASM Scoring guidelines [62]. A second pass by a trained human scorer was done to review and edit sleep staging following recommendations from the scoring software. Interclass correlation coefficients between human edited and automatically scored files were calculated for sleep stage times and the coefficient was > 0.8 overall for all files. Sleep scoring yielded the following variables: total sleep time (TST), sleep efficiency (SE), sleep onset latency (SOL), wake after sleep onset (WASO), time in each sleep stage (N1, N2, N3, REM), number of awakenings, total stage shifts, and number of arousals in REM and Non-REM (stages N2 and N3 combined). A discrepancy score between subjective and objective sleep times was calculated for TST, WASO, and SOL, by subtracting PSG values from the sleep diary values for each participant (subjective minus objective). The ORP data as well as average EEG power values for each band were provided by Cerebra Medical Inc on the sleep-scored edited files. EEG measured power was calculated by averaging the squared microvolts of power over the entire sleep file (from lights out to lights on) for each band (delta = 0.33–2.33 Hz, theta = 2.67–6.33 Hz, alpha = 7.33–12 Hz, sigma = 12.33–14.00 Hz, beta-1 = 14.33–20.0 Hz, beta-2 = 20.3–35.00 Hz, beta = average of beta 1 and beta 2) and separately for each EEG channel of the Prodigy equipment: left-frontal and right-frontal EEG channels. Only sleep diary and PSG/EEG data from the second night of recordings which preceded the afternoon of in-lab performance assessment were analyzed.

All variables were tested for violations of normality as well as outliers. Measures violating normality were square-root or log transformed and were tested again for normality. Outliers were identified by box-plots and by individual means greater than +/− 3 SD from the group mean.

Independent sample *t*-tests were used to compare good sleepers and individuals with insomnia symptoms for measures of mood (VAS-M, PANAS, STAI-State), sleepiness (SSS), affect (DASS), anxiety (STAI-Trait), sleep history (ISI, PSQI), sleep architecture, EEG band powers, and ORP values. A two-way ANOVA was conducted for analysis of response time, accuracy and intensity ratings on the Face Categorization and Intensity Rating Task, with between-subjects as Group (GS, INS) and within-subjects as Face (Happy, Sad, Angry, Fearful). For the Face-word Emotion Stroop Task, a three-way ANOVA with the between-subject factor of Group (GS, INS) and within-subject factors of Congruency (congruent, incongruent) and Face (Happy, Sad, Angry, Neutral) was conducted for response times. Equality of variance was examined using Levene’s test and violations were corrected using the Welch–Satterthwaite adjustment. For tests of sphericity, violations were corrected using the Greenhouse-Geisser correction.

An exploratory analysis of the interaction between group (GS, INS) and sleep on behavioural performance was conducted using a moderation regression model in PROCESS 3.3 macro for SPSS [63]. For regressions violating heteroscedasticity, heteroscedasticity-consistent standard errors using Hinkley’s method [64] were employed in consideration of the small sample size [65]. For the moderation model, predictors were examined independently and included subjective sleepiness, TST, time in Stage 2 sleep, time in SWS, time in REM, ORP Stage 2 sleep, ORP SWS, ORP REM, ORP NREM, PSQI, ISI, and beta power averaged over the two bands (beta-1 and beta-2). The moderation term was group (GS, INS). Further investigation of significant interactions was followed by a simple effects analysis using a bivariate correlational analysis for each group to determine how relationships between sleep and emotion processing outcomes might differ for each group.

#### Post-hoc Analysis of Poor Sleepers

Because individuals with insomnia do not sleep poorly on every night and previous research has suggested that differences in cognitive functioning occur after a night of poor or short sleep (e.g., [5,6,66]), a secondary post-hoc analysis of sleep and performance on emotion processing tasks was conducted to compare the good sleeper controls to the subset of individuals with insomnia symptoms that were confirmed to have a night of poor sleep prior to performance assessment (*n* = 8). Good and poor nights were judged using multiple variables including TST, SOL, SE, and WASO, from the two nights of PSG recording as well as sleep diary data.

## 3. Results

### 3.1. Participant Characteristics

Group comparisons of participant characteristics revealed that the INS group compared to the GS group had significantly greater complaints of insomnia severity and poor historic sleep quality indicated by greater scores on the ISI (*t*(18.20) = −6.65, *p* < 0.001), and PSQI (*t*(25) = −11.80, *p* < 0.001). No differences in affect were observed on the DASS subscales or for STAI-trait (Table 1).

### 3.2. Sleep Comparisons by Group

The INS group (*n* = 15) overall reported less TST on the sleep diary (*t*(24) = 3.61, *p* < 0.001), but differences in PSG-measured TST were not observed. No other differences in objective and subjective sleep parameters were observed (Table 2). For quantitative EEG measures of sleep, the INS group did not significantly differ in average power in any band across the night and an examination of group differences in ORP values did not find any differences in sleep depth (Table 3).

### 3.3. State and Psychomotor Vigilance Comparisons by Group

The INS group (*M* = 2.64, *SD* = 0.84) reported being significantly sleepier on the Stanford Sleepiness Scale than the GS (*M* = 1.87, *SD* = 0.64), *t*(27) = 2.81, *p* = 0.009. The INS group (*M* = 32.00, *SD* = 8.88) did not significantly differ from GS (*M* = 31.43, *SD* = 5.67) in positive state affect, nor did the INS group (*M* = 12.87, *SD* = 3.56) differ from GS (*M* = 12.79, *SD* = 6.58) in negative state affect. Further, the INS group (*M* = 33.67, *SD* = 7.78) did not differ in state anxiety from GS (*M* = 33.00, *SD* = 6.58). No evidence for differences between INS and GS groups was observed for psychomotor vigilance performance. Specifically, the INS (*M* = 247.16, *SD* = 30.47) and GS group (*M* = 240.81, *SD* = 51.02) did not differ in response time. There was no difference in number of lapses for the INS group (*M* = 0.36, *SD* = 0.50), compared to GS group (*M* = 0.40, *SD* = 0.74), nor the standard deviation of response time between INS (*M* = 51.02, *SD* = 24.24) and GS groups (*M* = 49.40, *SD* = 28.57). There was no observed difference in average RT for the INS group (*M* = 193.65, *SD* = 18.68) compared to GS (*M* = 190.71, *SD* = 19.17) for the fastest 10% of trials, and no difference in the slowest 10% of trials between INS (*M* = 344.26, *SD* = 72.34) and GS (*M* = 330.16, *SD* = 64.94) groups.

### 3.4. Emotion Processing Performance: Face-Emotion Categorization and Intensity Rating Task

The descriptive statistics for performance on both emotion tasks by group are presented in Table 4. The results from the analysis for response time, accuracy and intensity rating differences on the FCI are presented as follows:

For response time on the FCI task, the ANOVA revealed a non-significant interaction between Face and Group and no main effect of Group, but there was a significant effect of Face on response time (*F*(2.10,56.78) = 25.95, *p* < 0.001, *η*^2^ = 0.490). Happy faces were classified more quickly than Angry (*t*(26) = −4.90, *p* < 0.001), Fearful (*t*(26) = −8.10, *p* < 0.001) and Sad faces (*t*(26) = −9.64, *p* < 0.001).

The Face x Group ANOVA for accuracy yielded a non-significant Face x Group interaction and no main effect of Group, but a significant main effect of Face emotion was observed (*F*(1.91,51.52) = 22.92, *p* < 0.001, *η*^2^ = 0.459). Post-hoc comparisons of accuracy of the different expressions revealed that all participants were more accurate for Happy faces than Angry (*t*(26) = 6.00, *p* < 0.000), Fearful (*t*(26) = 7.68, *p* < 0.001), and Sad faces (*t*(26) = 7.25, *p* < 0.001), and more accurate for Angry faces than Fearful faces (*t*(26) = 4.72, *p* < 0.001).

For intensity ratings on the FCI, there was also a significant effect of Face, *F*(2.12,57.11) = 13.75, *p* < 0.001, *η*^2^ = 0.337, and again, no significant effect of Group, or significant Group x Face interaction. Post-hoc comparisons between Faces revealed that all participants rated Angry faces as more intense than Happy (*t*(26) = 3.20, *p* = 0.020) and Sad faces (*t*(26) = 4.92, *p* < 0.001), and also rated Fearful faces as more intense than Happy (*t*(26) = 4.64, *p* < 0.001) and Sad faces (*t*(26) = 6.60, *p* < 0.001).

In summary, there were no group differences observed for response time, accuracy and intensity ratings on the FCI. Overall, participants were faster and more accurate for Happy faces compared to other face-types. Additionally, all participants found Angry and Fearful faces as more intense than Happy or Sad faces.

### 3.5. Emotion Processing Performance: Face-Word Emotion Stroop

For the EST, the three-way interaction for response time was not significant, nor were the two-way interactions of Group x Face, Group x Congruency, or Congruency x Face. There was no main effect of Group. There was a main effect of Congruency on response time, *F*(1,27) = 155.31, *p* < 0.001, *η*^2^ = 0.852, and post-hoc pairwise comparisons found that all participants were slower for incongruent trials compared to congruent trials, *t* = −12.46, *p* < 0.001. There was also a significant main effect of Face on response time, *F*(3,34) = 49.04, *p* < 0.001, *η*^2^ = 0.879; post-hoc comparisons revealed that participants were significantly faster for Happy faces compared to Angry (*t*(26) = 9.90, *p* < 0.001), Sad (*t*(26) = 9.95, *p* < 0.001), or Neutral faces (*t*(26) = 10.52, *p* < 0.001). In summary, all participants were slower for incongruent trials compared to congruent trials and were faster for trials with Happy target faces than any other target faces and there were no detectable differences in response times between individuals with INS and GS.

Response time differences for the distractor-words on the EST were also examined. The three-way interaction between Group, Distractor, and Congruency for response times for distractors was non-significant. The two-way interactions between Group and Congruency, and Group and Distractor were also non-significant. There was a significant interaction between Congruency and Distractor for response time on the Emotional Stroop Task, *F*(3,81) = 30.80, *p* < 0.001, *η*^2^ = 0.533. A post-hoc analysis of distractors at the level of incongruent trials found that all participants were significantly slower for incongruent trials with a Happy distractor than Angry (*t*(26) = 4.65, *p* < 0.001), Neutral (*t*(26) = 5.37, *p* < 0.001) or Sad (*t*(26) = 4.53, *p* = 0.001) distractors (see Table 4). No significant main effect of Group was observed. The distractor word of “Happy” slowed response times more than any other distractors for incongruent trials and the INS group did not differ from GS in response time.

### 3.6. Post-hoc Analysis of Insomnia Subgroup with a Night of Objectively Poor Sleep

The post-hoc analysis comparing the INS group with a night of poor sleep preceding the afternoon of task performance (*n* = 8) and good sleepers (*n* = 15) revealed that this INS subgroup differed in sleep the night before testing. Specifically, the INS group with a night of poor sleep subjectively reported less total sleep time, *t*(19) = 3.77, *p* = 0.001 and differences in objective sleep architecture were found in less total sleep time (*t*(19) = 3.68, *p* = 0.006), less time in N2 sleep (*t*(19) = 2.63, *p* = 0.017), less time in REM sleep (*t*(19) = 2.99, *p* = 0.007), a smaller portion of time in REM, *t*(19) = 2.24, *p* = 0.038, and less time in Non-REM sleep overall (*t*(19) = 3.23, *p* = 0.004) than GS. This INS subgroup also trended towards greater power in the left-frontal sigma band, *t*(21) = −1.96, *p* = 0.064, and left-frontal beta-1 band, *t*(21) = −2.07, *p* = 0.052, compared to the GS group.

Some evidence for accuracy deficits in the expected direction were observed for this INS subgroup with a night of poor sleep. The ANOVA analysis of FCI accuracy with the INS subgroup with a night of poor sleep (*n* = 8) revealed a trending effect of Group in the direction of the hypothesis (using a 1-tailed test), *F*(1,21) = 2.90, *p* = 0.052, such that the INS group with a night of poor sleep had a trend for poorer accuracy overall on the FCI compared to GS. No other effects were observed in behavioural performance for this post-hoc subgroup analysis.

### 3.7. Moderation Analysis of Sleep Quality and Emotion Processing by Group

Overall, significant interactions between Group and beta EEG activity and follow up simple slopes analysis found that greater beta EEG activity at frontal sites was associated with greater intensity ratings for emotional faces of Happy, Fearful and Sad (trending), for the insomnia group. For the good sleepers, greater beta EEG activity was associated with poorer accuracy identifying Happy faces, and lower intensity ratings for Happy, Angry, Fearful (trending), and Sad faces. In addition, for good sleepers, greater sleepiness levels were associated with poorer accuracy for Fearful faces, and less time in slow wave sleep was associated with greater intensity ratings of Angry faces. These findings are reported in detail below.

**Figure 2:**
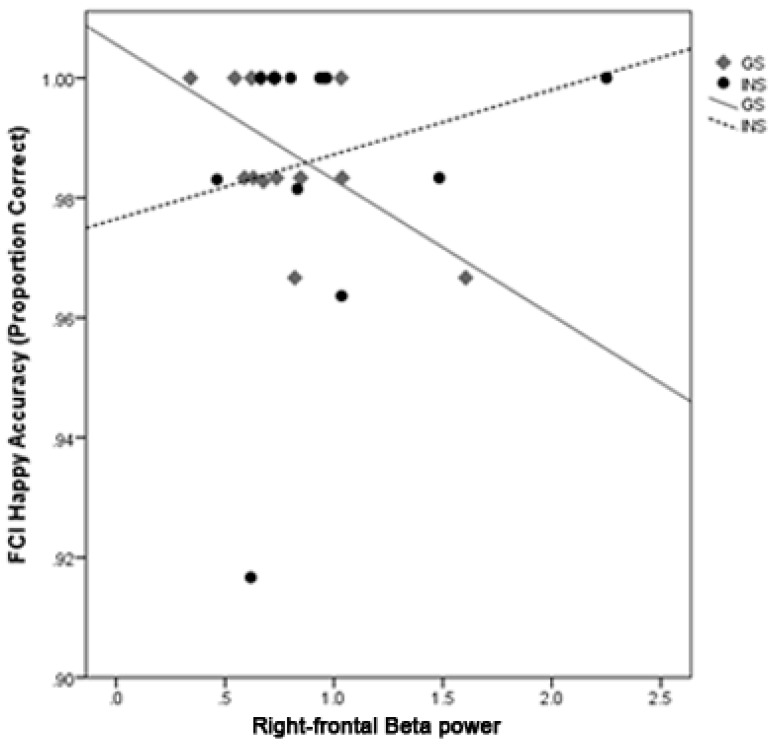
The correlations between right-frontal beta power and accuracy for Happy faces on the FCI for GS and INS groups. A night of greater right-frontal beta activity was associated with lower accuracy for the GS group.

**Figure 3:**
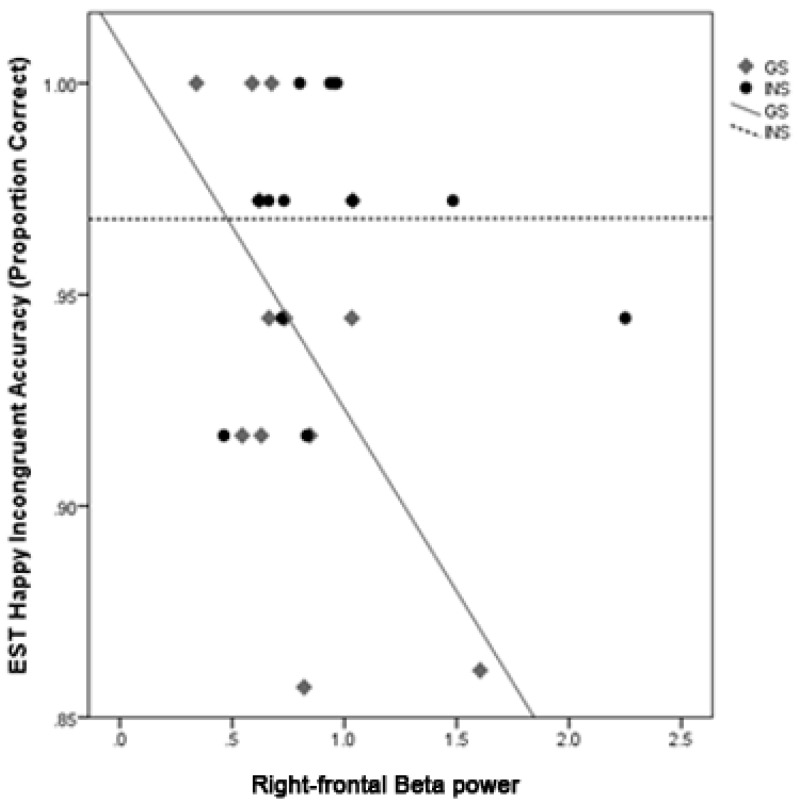
The correlations between right-frontal beta power and accuracy for incongruent Happy face trials on the Emotional-Stroop for GS and INS groups. A night of greater right-frontal beta activity was associated with lower accuracy for Happy faces on incongruent trials for the GS group.

**Accuracy.** There was a significant interaction between Group and right-frontal beta on accuracy for Happy faces on the FCI, *b* = 0.033, *t* = 2.83, *p* = 0.010. Simple slopes analyses for each Group revealed a significant negative relationship between right-frontal beta and accuracy for Happy faces for good sleepers, *b* = −0.02, *t* = −3.99, *p* < 0.001, but not for the INS group, *b* = 0.01, *t* = 1.04, *p* = 0.309 (Figure 2). For the EST, there was a significant interaction between Group and right-frontal beta on accuracy for incongruent Happy trials, *b* = 0.03, *t* = 3.00, *p* = 0.006. Simple slopes analysis of each Group revealed a significant relationship between greater right-frontal beta power and poorer accuracy for Happy faces on incongruent trials for good sleepers, *b* = −0.08, *t* = −3.77, *p* = 0.001, but no relationship was found for the INS group, *b* = 0.00, *t* = 0.01, *p* = 0.996 (Figure 3). Due to the lower variability in accuracy performance for Happy faces across participants, these relationships are interpreted cautiously.

**Figure 4:**
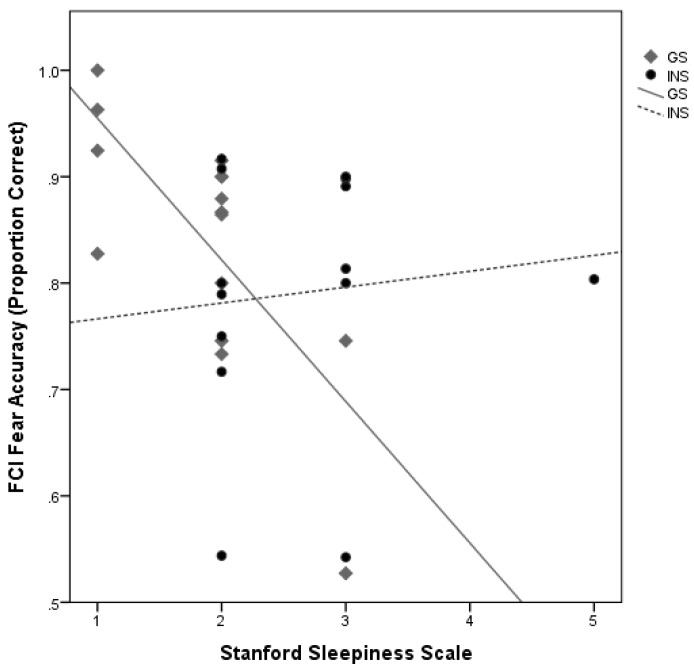
The correlation between Stanford Sleepiness Scale and accuracy for Fearful faces on the FCI for GS and INS groups. Sleepiness levels were negatively associated with Fearful accuracy for GS but not the INS group.

There was a significant interaction between Group and scores on the Stanford Sleepiness Scale on accuracy for Fearful faces on the FCI, *b* = 0.15, *t* = 2.89, *p* = 0.008. The simple effects analysis of each Group found that sleepiness scores were negatively associated with Fearful accuracy for good sleepers, *b* = −0.13, *t* = −3.08, *p* = 0.005, but not significantly associated in the INS group, *b* = 0.01, *t* = 0.55, *p* = 0.590 (Figure 4).

**Figure 5:**
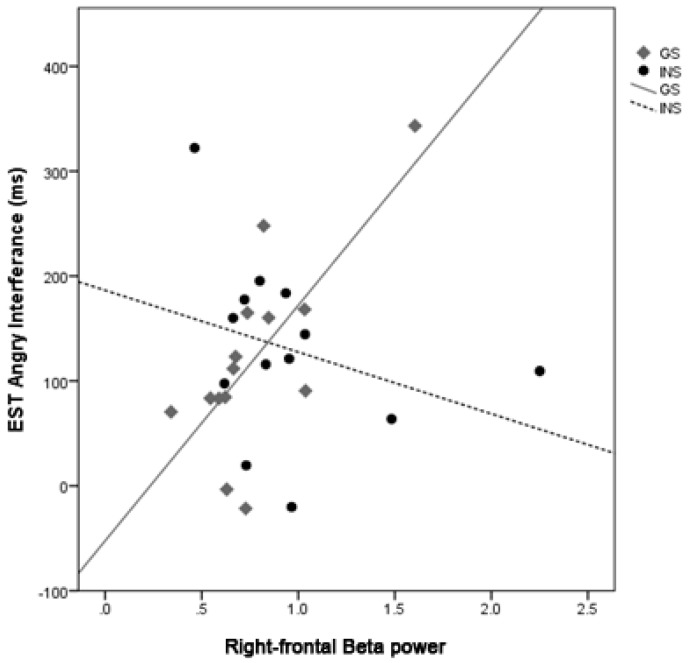
The correlations between right-frontal beta power and the interference effect for Angry trials on the Emotional-Stroop for GS and INS groups. A night of greater right-frontal beta power was associated with greater interference on Angry trials for the GS group.

**Angry interference.** There was significant interaction between Group and right-frontal beta, *b* = −283.27, *t* = −4.38, *p* < 0.001, on the interference effect during Angry trials. The simple slopes analysis for each Group revealed a significant positive correlation between right-frontal beta and Angry interference scores for the good sleepers, *b* = 224.40, *t* = 5.31, *p* < 0.001, but no significant relationship for the INS group, *b* = −58.87, *t* = −1.20, *p* = 0.242 (Figure 5).

**Figure 6:**
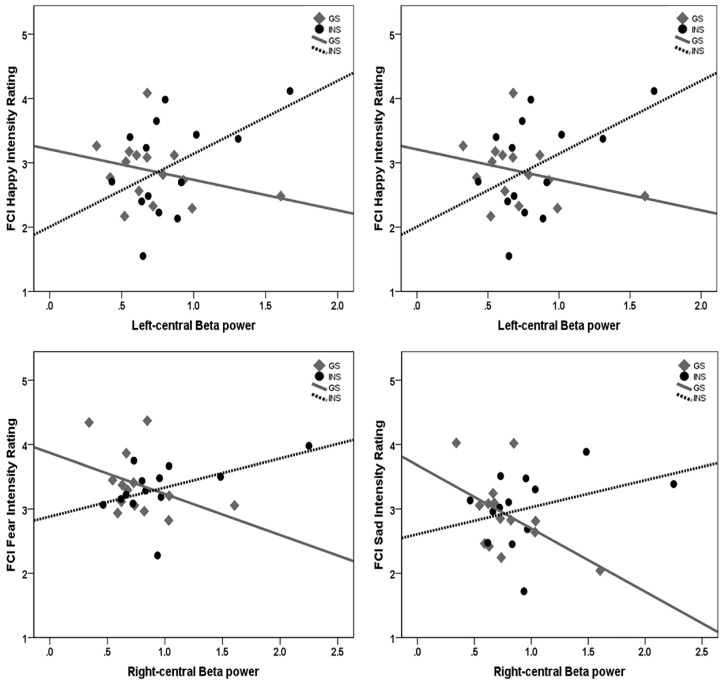
The correlation between frontal beta power and intensity ratings on the FCI for GS and INS groups. Greater frontal beta power was associated with greater intensity ratings of Happy, Fearful and Sad (trending) faces for the INS groups.

**Intensity ratings.** For intensity ratings for Happy faces on the FCI, there was a significant interaction between Group and left-frontal beta power over the night, *b* = 1.61, *t* = 4.16, *p* < 0.001. Simple slopes for each Group revealed a significant negative relationship between left-frontal beta and Happy intensity ratings for good sleepers, *b* = −0.47, *t* = −2.32, *p* = 0.029, but a significant positive relationship for the INS group, *b* = 1.13, *t* = −3.46, *p* = 0.002 (Figure 6). The interaction between Group and right-frontal beta power on intensity ratings for Happy faces was also significant, *b* = 1.12, *t* = 3.19, *p* = 0.004; there was a significant relationship between greater right-frontal beta and greater Happy intensity ratings for the INS group, *b* = 0.76, *t* = 4.22, *p* < 0.001.

There was also a significant interaction between Group and right-frontal beta power on intensity ratings of Angry faces, *b* = 0.98, *t* = 3.15, *p* = 0.004. A simple slopes analysis of each Group revealed a significant relationship between greater right-frontal beta and lower Angry intensity ratings for good sleepers, *b* = −0.99, *t* = −3.81, *p* < 0.001, but no significant relationship was found for the INS group, *b* = −0.01, *t* = −0.08, *p* = 0.941 (see Figure 6). There was also a significant interaction between Group and time in N3 sleep on intensity ratings of Angry faces, *b* = 0.01, *t* = 2.97, *p* = 0.007. A simple slopes analysis for each Group revealed a significant negative relationship between time spent in N3 sleep and Angry face intensity ratings for good sleepers, *b* = −0.01, *t* = −2.99, *p* = 0.006, but no relationship was detected for the INS group, *b* = 0.004, *t* = 1.39, *p* = 0.177.

There was a significant interaction between Group and right-frontal beta on intensity ratings for Fearful faces, *b* = 1.09, *t* = 2.97, *p* = 0.007. The analysis of simple effects for each Group found a significant positive relationship between right-frontal beta and Fearful intensity ratings for the INS group, *b* = 0.45, *t* = 6.12, *p* < 0.001, and a trend for a negative relationship for good sleepers, *b* = −0.64, *t* = −1.77, *p* = 0.089 (Figure 6). There was also a significant interaction between Group and left-frontal beta power on intensity ratings for Fearful faces, *b* = 0.96, *t* = 2.33, *p* < 0.028; analysis for each Group revealed a significant relationship between greater left-frontal beta and greater Fearful intensity ratings for the INS group, *b* = 0.67, *t* = 3.40, *p* = 0.002, but no relationship between these variables for good sleepers, *b* = −0.31, *t* = −0.84, *p* = 0.411.

Finally, there was a significant interaction between Group and right-frontal beta power on Sad intensity ratings, *b* = 0.96, *t* = 2.33, *p* < 0.028 (Figure 6). The simple slopes analysis for each Group revealed a significant relationship between greater right-frontal beta and lower Sad intensity ratings for good sleepers, *b* = −0.98, *t* = −3.03, *p* = 0.006, but a positive relationship between right-frontal beta and Sad intensity ratings approaching significance for the insomnia group, *b* = 0.42, *t* = 2.04, *p* = 0.053.

## 4. Discussion

In the current study, emotion processing tasks were examined after a night of PSG recording in order to determine how sleep contributes to next-day processing of emotionally expressive faces in insomnia and good sleepers. The insomnia group was found to be sleepier during the afternoon of performance testing and reported shorter sleep durations than good sleepers despite a lack of evidence for poor sleep quality in PSG measures. Individuals with insomnia did not show any evidence of disturbed mood or affect and no evidence for general impairment in emotion processing performance was found. Greater beta EEG power during the night was associated with greater intensity sensitivity for emotional happy, fearful and a trend for sad faces for individuals with insomnia symptoms. For good sleepers, greater beta EEG power in sleep was associated with poorer perceptual accuracy for happy faces with and without the presence of emotional distractors as well as worse inhibitory control for distracting emotional words when detecting angry faces. In good sleepers, greater beta EEG power was also associated with lower intensity ratings for happy, angry and sad faces and a trend for fearful faces. For the good sleepers but not the insomnia group, greater sleepiness was associated with poorer accuracy for fearful faces, and less time in slow wave sleep was associated with greater sensitivity to angry faces (based on intensity ratings).

### 4.1. Hyperarousal Differentially Affects Emotion Processing in Good Sleepers and Insomnia

The diverging directions of the relationship between greater frontal beta EEG power during sleep/wake and performance for insomnia and good sleeper groups suggests that hyperarousal (i.e., greater beta EEG power) may be associated with heightened salience processing of emotional face expressions for individuals with insomnia symptoms for some emotions, but in contrast, both blunts sensitivity and leads to emotion processing performance deficits in good sleepers. This points to the possibility for a potential maladaptation or alteration in socioemotional salience processing directly linked to neurophysiological activity at sleep onset and throughout sleep for individuals with insomnia. Possible explanations for these contrasting findings are that for good sleepers, high levels of beta EEG activity may represent a rare night of light, non-restorative sleep (perhaps due to environmental factors), which taxes cognitive resources for next-day functioning. Whereas, for the insomnia group, elevated beta EEG power may represent activity in emotional processing regions throughout sleep that affects next day emotional processing, or alternatively, maladaptation in emotion reactivity during the waking state in insomnia may result in both greater sensitivities to emotional faces and a predisposition for hyperaroused sleep.

Neurophysiological examination of both GABA and regional differences in activity in sleep and wake in insomnia provide evidence that hyperarousal in insomnia may be specific to altered activity in emotion processing regions. GABA is the primary neurotransmitter related to inhibition of the CNS and plays a critical role in sleep initiation, sleep onset and sleep maintenance [67]. Critically, several studies have identified lower GABA levels overall in insomnia [68,69], but one study by Plante and colleagues [70] specifically identified lower levels of morning GABA in the anterior cingulate cortex which is a key structure in emotion information processing including the processing of emotion faces. There has also been imaging evidence for smaller differences in the activity between wake and sleep in regions of affect and face processing, including the left fusiform gyrus and posterior cingulate cortex [22]. Nofzinger and colleagues [71] also found that greater objective or subjective time spent awake throughout the night (i.e., difficulties with sleep maintenance and arousability) were associated with increased activity in the anterior cingulate cortex, and regions associated with emotional awareness, anxiousness and fear (temporal poles). Event-related potential analysis of auditory stimulus processing during sleep for individuals with insomnia has also revealed markers of sustained/uninhibited information processing (e.g., [34,72]). Thus, hyperarousal may represent either the continuous activation or a failure to inhibit the activation of emotional information processing regions during sleep in insomnia, and this may ultimately lead to alterations in the functioning of salience processing during wake. Further research should investigate which regions appear to be active/uninhibited in insomnia sleep to elucidate if hyperarousal or sustained activity exists within emotional regions.

Another possible consideration is that socioemotionally sensitive or reactive individuals are predisposed to nights of hyperarousal. Kalmbach et al. [73] suggested that insomnia may be a condition of dysregulated cognitive-emotional reactivity to stress, ultimately leading to increased reactivity to sleep and thereby, longer periods of sleep onset, greater pre-sleep ruminations and worry, and sleep disruption (i.e., insomnia symptomology). This is not a novel concept, as established models of insomnia have also pointed to maladaptive cognitions and emotion-reactivity, as etiological and pathological qualities of insomnia (see [4] for a review). Intervention with stimulus control and relaxation training for individuals with insomnia has been shown to lead to a reduction in elevated beta EEG activity at sleep-onset [74], supporting the notion that elevated beta may represent maladaptive cognitive-emotional reactivity and conditioned arousal. It is possible that dysregulated or maladaptive cognitive-emotional reactivity in insomnia extends beyond the processing of stressors and negatively conditioned sleep stimuli into the processing of emotional stimuli such as faces as well. A link between hyperarousal and altered waking cognitive-emotional reactivity might be drawn out by EEG examination during wake; increased physiological and subjective arousal during wake in reaction to sleep and non-sleep related emotion stimuli reported in individuals with insomnia [23] has already demonstrated an increase of reactivity to emotion stimuli as a potential feature of this clinical group, but has not been linked to hyperarousal. If hyperarousal in insomnia manifests as a condition of altered cognitive-emotional reactivity during wake, then inconsistent findings in waking hyperarousal [45] may be due to physiological measures predominantly taken while research participants are in resting states rather than in response to emotional stimulation and/or task engagement.

A final possible consideration is that the chronicity and regularity of poor sleep and/or hyperarousal in insomnia might be an important contributing factor to emotion processing differences in insomnia and good sleepers. It has been suggested that sleep plays an integral role in restoring or resetting emotional circuits to correctly react and process emotional information the following day [75]. Thus, repeated nights of poor sleep and/or neurophysiological engagement (hyperarousal insomnia) might disrupt the restorative properties of sleep. In turn, chronic disruption could lead to structural/functional changes in emotional processing neurological regions (e.g., [22,76]) and to abnormal or maladaptive sensitivities to emotional information during wake, beyond that which is seen after a rare night of light or poor sleep in good sleepers. Some evidence suggests that multiple nights of poor sleep is related to greater waking impairment in cognitive functioning (e.g., [8,66]). However, the presence of repeated or consistent nights of hyperaroused sleep in insomnia and the impact that nights of hyperaroused sleep have on waking cognitive functioning has not been well investigated. Nonetheless, several findings of hyperarousal during insomnia sleep [32,33,34,35,36,37,38,39,40,41,42] suggested that it could be a consistent pathological quality of sleep in this clinical population. Therefore, future efforts should be made to longitudinally examine hyperarousal sleep and the impact that it has on waking functioning.

### 4.2. Sleep and Emotion Processing Group Differences

Except for diary reports of less total sleep time, no significant differences in sleep between good sleepers and individuals with insomnia in the full sample were observed. The finding that individuals with insomnia symptoms subjectively reported less total sleep time than good sleepers in their sleep diaries, despite no observable differences in PSG measure of total sleep time, is consistent with the existing literature (e.g., [77,78,79,80]). We identified that eight of the fourteen insomnia participants had a night of poor sleep, while six of the fourteen insomnia participants had a good night of sleep. And, only six of the fourteen individuals with insomnia symptoms reported clinical levels of insomnia on the ISI. Due to the small sample size, all insomnia participants were included together for the primary analyses. Having a small sample, with a mixed and predominantly sub-clinical insomnia group likely affected sensitivity to find the expected group differences in gross sleep architecture, quantitative EEG differences, as well as emotion processing performance. The novel ORP measure (which captures sleep-depth and arousability) may be more suitable to characterizing the sleep of individuals with insomnia that have been specifically identified as having difficulty remaining and/or maintaining sleep and whom have frequent awakenings (i.e., issues with sleep depth and arousability).

In the insomnia group that had a night of poor sleep, we observed a trending effect for greater sigma and beta-1 band power, as well as differences in sleep architecture: less time in Stage 2 and REM sleep. These differences have been previously reported in other studies of sleep in insomnia [31,36,41]. The loss of Stage 2 and REM may be attributable to fewer sleep cycles occurring for those with a night of poor sleep, as both Stage 2 and REM become more prominent later in the night [81]. Greater power in beta and sigma has been suggested to indicate a simultaneous activation of sleep and wake-promoting activity in insomnia sleep [41]. Krystal and colleagues [36] also found elevated sigma and beta activity but only for a subjective phenotype of insomnia and not an objectively poor sleeping insomnia group. Here, we found some evidence for elevated sigma and beta in a small insomnia group objectively identified to have a night of poor sleep.

Two recent reports of impairment in sensitivity for emotionally expressive face stimuli [24,25] for the insomnia group were not replicated here. Kyle and colleagues found individuals with insomnia who had greater anxiety and depressive symptoms had a greater blunting in the intensity ratings of emotional faces [24]. Cronlein and colleagues found impaired accuracy performance for an insomnia group who were identified to have objective nights of poor sleep, but also found similar effects in a group with sleep apnea [25]. Therefore, it is possible that because the current sample failed to show evidence of objective sleep impairment, and participants were recruited based on an absence of affective disturbance (i.e., anxiety or depression), we were unable to detect overall differences in emotion processing performance from good sleepers. It is also possible that some participants with insomnia may show resiliency or compensation with respect to the impact of insufficient sleep on waking performance; these individual differences could be investigated in future research. However, in support of Cronlein and colleague’s findings, some evidence for impaired accuracy recognizing faces after a night of poor sleep in insomnia was observed in the current study. In light of the findings by Kyle and colleagues and the current findings of a relationship between beta EEG power during sleep and altered salience processing in insomnia, further research should be conducted on the interplay between hyperarousal during sleep and both affective disturbance and salience processing during wake. It is also suggested that poor sleep in insomnia may lead to performance deficits in emotional face perception, similarly to that which has been reported after nights of experimental sleep deprivation [82,83,84,85], which might also contribute to socioemotional impairment. The extent and type of emotional impairment after nights of poor sleep and hyperarousal in insomnia requires further investigation.

### 4.3. Limitations, Conclusions and Future Directions

The sample was small and included a predominantly subclinical insomnia group with a mixture of good and poor nights of sleep. Thus, the current findings should be interpreted with caution. Future investigations of the effects of sleep (and hyperarousal) in insomnia on waking function must include larger samples to better identify effects, as well as to account for variability in sleep, insomnia subtypes, and the presence or absence of hyperarousal. The sample in the study was also predominantly a convenience sample from the university student body, and as such, the sample was predominantly women and young adults. Therefore, there was no opportunity to examine the effects of sleep and emotion processing between sex, and it is cautioned that any particular effects observed here may be generalizable only to women. Importantly, the findings reported here, or even lack of effects, could be attributable to the skew of more women in the sample, and thus further studies should be done in men. The findings also may not generalize to individuals outside the age range examined for the current study, including older adults for whom insomnia is more prevalent.

Sleep was recorded in participants’ own homes in order to increase the ecological validity of the findings which is particularly useful with the insomnia sample who tend to sleep well away from their home environment. However, without strictly enforced bed times, a large portion of the good sleeper participants were lost to sleep restriction, and the insomnia group may have employed strategies such as delaying bedtime which may have contributed to an absence of observable differences in sleep efficiency between groups. In addition, without constant monitoring, some elements of the data, such as data from an EEG channel or sleep diaries, were lost for some participants. A very important limitation to the ambulatory PSG employed in this study was the absence of the ability to detect and rule out sleep apnea and restless leg syndrome. Both conditions were screened for during the intake process by interview and by questionnaire; however, it is possible that participants who are unaware of underlying medical sleep conditions entered the study. Another lack of control in the home environment was that participants could turn the equipment on well before the intention to go to sleep (e.g., reading) despite instruction to record from lights off to on. Thus, measures of wake time, sleep onset latency, and sleep efficiency may not be precise for all participants. Better home monitoring may be achieved with increased controls and measures of compliance as well as an in-lab screening night. A strength of the current study was that performance was assessed on the day immediately following the PSG recording; future studies must endeavour to investigate the impact of the preceding night of sleep on waking performance.

Hyperarousal in the current study was measured by investigating beta EEG during the entire sleep period, including sleep onset, wake time, and non-REM and REM sleep stages together. Higher frequency EEG in the gamma band which has also been associated with hyperarousal was not investigated in the current study because of hardware filters imposed on the data, but should be examined in future studies. In addition, the beta EEG reported in the current study was not separated by wake and sleep stages. While some studies have reported hyperarousal specific to stages of sleep [36,38,41], others have noted the presence of hyperarousal in pre-sleep wake and at sleep onset as well (during the effortful intention to fall asleep; [74,86,87], and some during both pre-sleep wake and sleep [35,42]. Because we had a heterogeneous sample of individuals with insomnia symptoms (i.e., not exclusively sleep onset or maintenance problems), we choose to sample the entire sleep/wake record for EEG analysis. Since groups did not differ in wake time, it is likely that at least some of the elevated beta EEG is coming from sleep, although future studies in larger samples of more homogenous insomnia subtypes may be able to better address the hyperarousal during wake and sleep states (or even transitions and arousals).

This study investigated the relationship between quantitative EEG measures of sleep and the next-day socioemotional processing of faces in individuals with insomnia symptoms and good sleepers. The contrasting associations between beta EEG activity and emotion processing for good sleepers and insomnia groups suggests that hyperarousal in insomnia may lead to waking consequences of altered salience processing, and more specifically, to heightened sensitivity to emotionally expressive faces. Given the limitations of the sample, further studies are warranted. The following research questions are recommended to be addressed by future research efforts: are emotion processing areas engaged during hyperarousal sleep; are the same areas engaged during hyperarousal in both good sleepers and individuals with insomnia; does hyperarousal have functional significance for good sleepers as well; finally, is hyperarousal associated with abnormal neurophysiological reactivity to emotional cues during wake? Clinically, the findings suggest that hyperarousal in insomnia relates to abnormal sensitivity to emotionally expressive faces, which could contribute to the reported experiences of poorer social functioning. Interventions which restore or curtail neurophysiological activity during sleep to normative levels may also serve to improve socioemotional functioning by restoring appropriate sensitivity to the emotional expressions of others. Further confirmation of the impact of hyperarousal and a night of poor sleep on emotion processing in insomnia could also inform possible treatments or inventions for the development of comorbid social deficiencies, anxiety and/or depression.

## Figures and Tables

**Table 1:** Group comparisons and means and standard deviations for historic sleep quality and trait affect.

	Good Sleepers (*n* = 14)		Insomnia (*n* = 15)				
	*M*	*SD*	*M*	*SD*	*t*	*df*	*Sig. (2-tailed)*
ISI	3.60	2.56	14.21	5.44	−6.65	18.20	0.001 **
PSQI	2.87	1.55	11.39	2.14	−12.16	26	0.001 **
DASS-Anxiety	2.60	2.29	3.71	4.34	−0.86	19.43	0.403
DASS-Depression	3.73	3.53	6.36	6.78	−1.29	19.28	0.211
DASS-Stress	6.00	4.17	8.71	5.80	−1.46	27	0.157
STAI-Trait	36.87	9.46	39.86	14.07	−0.68	27	0.505

*Note.* ** = significant at *p* < 0.001.

**Table 2:** Group comparisons and means and standard deviations for PSG sleep, sleep diary, and the discrepancy between objective and subjective sleep.

	Good Sleepers (*n* = 14)		Insomnia (*n* = 15)				
	*M*	*SD*	*M*	*SD*	*t*	*df*	*Sig. (2-tailed)*
PSG Parameters							
TST	432.82	33.42	393.42	78.27	1.68	15.99	0.113
SE	93.39	2.65	89.74	10.28	1.24	13.48	0.236
SOL	12.39	7.70	16.69	14.79	−0.96	25	0.347
WASO	19.43	10.18	28.08	36.89	−0.82	13.69	0.428
Wake Time	29.83	21.07	40.28	39.20	−0.87	25	0.392
N1 Time	46.94	19.74	42.92	17.84	−0.10	24	0.923
N2 Time	232.90	47.52	203.01	45.81	1.66	25	0.109
N3 Time	80.48	33.76	88.69	30.30	−0.66	25	0.514
REM Time	74.22	18.20	62.42	33.88	1.14	25	0.265
NREM Time	362.65	36.95	336.72	53.71	1.47	25	0.154
Number of Awakenings	16.07	6.04	14.69	7.86	0.51	25	0.612
Total Stage Shifts	129.21	24.95	126.31	35.12	0.25	25	0.805
Number of Arousals in REM	12.36	7.41	12.38	8.09	−0.01	25	0.993
Number of Arousals in Non-REM	73.43	19.64	76.15	33.38	−0.26	19.13	0.801
Sleep Diary							
TST	475.85	50.67	389.23	70.14	3.61	24	0.001 *
SOL	12.89	6.60	27.69	35.51	−1.48	12.83	0.163
WASO	6.38	8.20	17.85	20.80	−1.64	11.33	0.128
Sleep Quality Rating (1–7)	4.54	1.51	3.92	0.95	1.24	20.29	0.228
Number of Awakenings	1.92	1.88	3.42	4.42	−1.08	22	0.291
Subject-Objective Discrepancy							
TST	32.38	31.95	−4.19	92.12	1.35	14.85	0.196
SOL	2.08	4.59	11.00	29.18	−1.09	12.59	0.297
WASO	−12.21	12.27	−14.35	43.42	0.20	13.76	0.847

*Note.* * = significant at *p* < 0.05.

**Table 3:** Group comparisons and means and standard deviations for quantitative EEG parameters of sleep.

	Good Sleepers (*n* = 14)		Insomnia (*n* = 15)				
Measure	*M*	*SD*	*M*	*SD*	*t*	*df*	*Sig. (2-tailed)*
Average EEG Power							
LF delta	227.26	132.69	208.15	85.48	0.45	26	0.660
RF delta	263.67	153.41	226.53	113.74	0.51	24	0.618
LF theta	13.87	4.78	13.82	3.78	0.03	26	0.975
RF theta	15.58	4.90	15.29	3.82	0.17	24	0.870
LF alpha	5.00	2.60	4.37	1.18	0.80	26	0.430
RF alpha	5.99	2.85	5.08	1.40	1.00	24	0.327
LF sigma	0.71	0.23	0.93	0.45	−1.68	27	0.104
RF sigma	0.85	0.29	1.09	0.49	−1.56	25	0.132
LF beta-1	0.87	0.27	1.04	0.40	−1.36	27	0.186
RF beta-1	0.95	0.30	1.17	0.50	0.51	25	0.181
LF beta-2	0.57	0.36	0.63	0.26	−0.54	27	0.594
RF beta-2	0.60	0.32	0.74	0.44	−1.08	25	0.279
LF beta Average	0.71	0.31	0.84	0.32	−1.10	27	0.277
RF beta Average	0.78	0.30	0.96	0.46	−1.27	25	0.216
ORP Measures							
ORP Non-REM	0.50	0.17	0.51	0.18	−0.23	27	0.905
ORP REM	0.67	0.30	0.74	0.40	−7.08	27	0.638
ORP N1	0.84	0.26	0.89	0.25	−1.06	27	0.609
ORP N2	0.53	0.18	0.54	0.17	−0.33	27	0.869
ORP N3	0.24	0.11	0.25	0.13	−0.18	26	0.883
R/L ORP Correlation	0.87	0.09	0.87	0.12	1.03	26	0.560
ORP Max During Arousal	1.99	0.21	2.00	0.18	−0.38	27	0.925
ORP Baseline Before Arousal	0.63	0.22	0.61	0.21	0.01	27	0.786
Number of Arousals with ORPMax -ORPBaseline > 0.5	45.27	16.95	48.50	26.92	0.66	27	0.886
ORP-9 Post Arousal	0.81	0.20	0.80	0.23	0.03	27	0.700

*Note.* LF = Left-frontal EEG channel. RF = Right-frontal EEG channel. ORP = Odd Ratio Product (values range from 0 = deepest sleep to 2.5 = wake/arousal).

**Table 4:** Means and standard deviations for measures from the Face Categorization and Intensity Rating Task (FCI) and the Face-word Emotion Stroop Task (EST).

	Good Sleepers (*n* = 14)		Insomnia (*n* = 15)		All	
	*M*	*SD*	*M*	*SD*	*M*	*SD*
FCI Response Time						
Happy	402.46	113.92	367.48	92.97	385.57	104.01
Angry	480.93	120.49	448.98	103.10	465.51	103.10
Fearful	496.34	115.62	488.87	87.28	492.73	101.17
Sad	487.78	128.46	460.18	89.03	474.46	110.13
FCI Accuracy						
Happy	0.99	0.01	0.98	0.03	0.98	0.02
Angry	0.90	0.08	0.90	0.08	0.90	0.08
Fearful	0.84	0.12	0.79	0.12	0.82	0.12
Sad	0.86	0.09	0.82	0.13	0.84	0.11
FCI Intensity Ratings						
Happy	2.87	0.49	2.96	0.75	2.91	0.62
Angry	3.30	0.51	3.23	0.48	3.26	0.48
Fearful	3.39	0.48	3.29	0.41	3.34	0.44
Sad	2.92	0.56	2.99	0.55	2.95	0.55
EST Interference (Faces)						
Happy	95.75	58.77	85.15	61.47	90.63	59.25
Angry	126.06	91.91	133.65	86.48	129.73	86.48
Sad	109.55	100.90	119.61	76.94	114.41	88.69
Neutral	137.52	95.87	122.94	101.22	130.48	96.99
EST RT Incongruent Trials (Distractors)						
Happy	1048.60	178.36	1029.85	162.81	1039.55	168.24
Angry	994.90	144.24	977.89	158.19	986.69	148.15
Sad	1000.10	180.46	956.92	148.14	979.25	164.18
Neutral	997.75	207.40	951.96	161.64	975.13	184.82
